# Preterm children quality of life evaluation: a qualitative study to approach physicians’ perception

**DOI:** 10.1186/1477-7525-10-122

**Published:** 2012-09-28

**Authors:** Marie-Ange Einaudi, Marie-Claude Simeoni, Catherine Gire, Pierre Le Coz, Sophie Condopoulos, Pascal Auquier

**Affiliations:** 1 Department of Neonatology, APHM, North University Hospital, Marseille, France; 2 Espace Ethique Méditerranéen, Aix-Marseille Univ, UMR 7268 - Anthropologie bio-culturelle, Droit, Éthique & Santé (ADÉS), APHM, Timone University Hospital, Marseille, France; 3 EA 3279 « Self Perceived Health Assessment Research Unit », Aix-Marseille Univ, Marseille, France; 4 Social Psychologist, CAMSP La Rose, Marseille, France

**Keywords:** Qualitative study, Focus group, Preterm children, Quality of life, Physicians’ perception, Ethical issues

## Abstract

**Background:**

While data for preterm children health-related quality of life are available, there are little data on the perception of health-related quality of life evaluation by physicians who manage preterm children, or its use in real life and decision making. The aim of this qualitative study is to highlight among physicians, themes of reflection about health-related quality of life in extremely preterm children (less than 28 weeks’ gestation).

**Methods:**

Focus groups at a French University Hospital with physicians who manage extremely preterm children: obstetricians, intensive care physicians, neonatal physicians and paediatric neurologists. The focus groups allowed the participants to discuss (drawing on their personal experience), three principal topics regarding the health-related quality of life of preterm children: representation, expectations in daily practice and evaluation method.

**Results:**

We included fourteen participants in the three focus groups. Many themes emerged from the focus groups: approaches for defining health-related quality of life and difficulties of utilization, the role that health-related quality of life should have in the system of care, the problem of standards and evidence-based decision making. Physicians had difficulties with taking positions regarding this concept. There were no differences by gender, age or seniority, but points of view varied by specialty and type of practice. Physicians who had longer specialized care for extremely preterm children were more sensitive to the impact of preterm complications on health-related quality of life.

**Conclusions:**

This study provides preliminary results about physicians’ perspective on the health-related quality of life of extremely preterm children. The themes emerged from the focus groups are classically described in other domains but not all in so clear a way (definition, interests and limits, ethical reflection). This approach was never developed in the field of prematurity with well-knowed consequences on quality of life. These results require to be confirmed on a larger representative sample. The themes and questions of this broad opinion survey will rest on the information issued from our preliminary interviews.

## Background

The consequences for extremely preterm children (EPC) (<;28 weeks’ gestation) are well known in terms of mortality and morbidity [1,2]. Interest is increasing in not only these traditional health indicators but also in the subjective representation of health and well-being, also known as health-related quality of life (HRQoL); the subjective evaluation of quality of life (QoL) is possibly as important for the patient as the traditional focus on morbidity and the prolongation of life [3,4]. HRQoL evaluation seems important because complications affect the daily basis of formerly EPC during early school age [5-7]. However, even if data on HRQoL for formerly EPC are available, they are limited because age groups are limited; indeed, if consequences on HRQoL seem more marked at early preschool age [8] than at the teenager [9] or the young adult [10,11], this report can be biased because of the mode of evaluation of the QoL which varies with the age of the subject and according to the methodology [3,4].

In chronic diseases, the perceptions of physicians of the HRQoL of their patients often differ from those of the patients and their families [8,12-14]. While HRQoL data are available, there are little data on the basic understanding and perception of quality of life issues by perinatologist physicians as well as the day-to-day experience of the patient or the role it plays in clinical decision-making [15-17].The need for quality data in this area is all the more important as these physicians are directly involved in the decisions taken with regard to neonatal resuscitation (even if these decisions take into account the families’ opinions) as well giving us a more stable platform for relaunching the public debate. What is the impact of the neonatal resuscitation in term of quality of life?

It would be relevant to highlight the opinions of perinatology experts regarding EPC HRQoL, and in particular physicians because they are the decision-makers. We report in a preliminary qualitative study the first step of the project. The aim was to produce themes of reflection and identify subjects that would imply study on a larger scale.

## Methods

Eligible participants were experts who manage EPC at the French University Hospital of Marseille, composed of four hospitals. Two of them are involved in the care of very preterm children (level 3 maternities), one in the long term follow up of the children; another hospital is involved in the long term follow up. The participants were defined as follows: more than 18 years old; both genders; having a minimal level of resident practice; involved in the care of the EPC (obstetricians, intensive care physicians, neonatal physicians, psychologists and paediatric neurologists); having agreed to participate in the study; registered on the list of the staff working in the University Hospital. The participants were selected by drawing lots stratified by the main clinical functions from the list. They were asked to complete a short socio-demographic and clinical characteristics questionnaire.

We used the qualitative method of focus groups (FGs) to generate data using the opinions collectively expressed by the participants [18-20], as it has been used in other contexts and more specifically with children [21].

Focus groups were designed to include at least one representative from each specialty. Confounding factors such as age, gender, function and practice were also considered; according to the hospitals, preterm children can be cared in departments of neonatal intensive care units where neonatologists work, and in mixed paediatric and neonatal intensive care departments (newborns and older children) where intensive care physicians work. The FG were organized according to the availability of the participants. A minimum of two FGs was planned, but the number was increased until a saturation of information was reached beyond which no new concepts were emerging from the FGs. Five new participants would be selected from new drawing lots.

Focus groups were audio-recorded and moderated by a well trained social psychologist (SC) using a discussion guide with guidelines and open-ended questions. The guide was based on an analysis of the literature and centred on the prioritised themes to be discussed: HRQoL representation, expectations in daily practice, HRQoL evaluation (i.e. Table 1). The psychologist supplemented the prepared questions with sub-questions that enabled the researchers to explore participants’ answers in greater detail. She encouraged equal participation from the whole group and encouraged people to engage with one another to create a dynamic discussion that would characterize the specificity of group conversations rather than individual interviews.

**Table 1:** Discussion guide

**Main themes**	**Sub- themes**	**Associated questions**	**Questions of relaunching**
HRQoL* representation	Perception	What does the HRQoL concept evoke for each of you?	What do you think about HRQoL and well-being measurements?
		How do you perceive this notion?	How would you define HRQoL as a concept?
			What do you consider the important dimensions?
			What is the time of relevance of such a concept?
	Knowledge	What knowledge do you have of its application in the field of health?	Do you know of tools for the evaluation of HRQoL?
			Do you think they are relevant? Which one? Why?
	Experience	What experience do you have with it in your practice?	Have you ever participated in a study of HRQoL?
			Have you already used a HRQoL questionnaire in your practice? Why? How?
			Do you assess the HRQoL of your patients? How? Why?
Expectations in daily practice of such a concept in extremely preterm children	Interests	What is your interest in the HRQoL concept?	What is the utility of its evaluation in your specific practice?
			What are the interests of the medical community towards such data?
			How could HRQoL evaluation change the way your practice for your patients?
	Limitations	What are the limitations of this concept and its evaluation?	Do you think that a standardised evaluation could potentially damage your intuitive evaluation of the well-being of your patients?
HRQoL evaluation	HRQoL evaluation in daily practice	Do you think every patient should be evaluated?	How could HRQoL assessment become a tool integrated into the care of preterm children in your daily practice?
		How could you use HRQoL evaluation in daily practice?	
		When do you think it would be important to make this evaluation?	What effects and consequences could its evaluation have in your relationship with your patients?
		Who should assess HRQoL?	What would you make of a “bad” HRQoL evaluation?
	Questions about the tool	What would be an ideal questionnaire?	What do you think of the existing questionnaires?
			How would you envision a standardized evaluation: number of questions, duration, kind of notation?
			What are the important dimensions of HRQoL to estimate?

*HRQoL: Health-related quality of life.

All the sessions were manually content-analysed by the social psychologist. The data were coded and classified by reviewing the transcribed discussions for categories, using the guideline questions as initial categories. Data relevant to the research questions were identified. Within categories, the different items were written up as statements. Relevant statements that did not fit into the predefined categories were assigned to new categories [19,20,22,23]. A second independent review of the data codes and categories was made by a physician (MAE). At the end, the coherence of this content analysis was confirmed by an external expert from the Self Perceived Health Assessment Research Unit (MCS). Opinions were compared between the participants’ characteristics (gender, age, experience, specialty and practice-type).

All participants provided informed consent. Anonymity and confidentiality of information were guaranteed. Ethical approval was obtained from French legal authority: the National Committee of Computing and Liberties.

## Results

### FG characteristics

Fifteen participants were contacted and 14 participated. The characteristics of participants are reported in Table 2. Two FGs were initially conducted; in the second, new information emerged with regard to the first FG, so we conducted a third FG during which saturation of information was obtained.

**Table 2:** **Demographic data and clinicalcharacteristics**

**Focus groups population**	**N=14**	**First Focus group N=4**	**Second Focus group N=6**	**Third Focus group N=4**
**Age** (years)	36.3 (24-62)	25.5 (24-27)	41 (28-62)	40.2 (31-53)
Female **gender**	10 (71.4%)	4 (100%)	3 (50%)	3 (75%)
**Experience with extremely pretermchildren** (years)	7.8 (1-36)	2 (1-4)	11.8 (3-36)	7.5 (2-10)
**Function:**				
Senior physicians	8 (57.1%)	0	4 (66.7%)	4 (100%)
Junior physicians	5 (35.7%)	4 (100%)	1 (16.7%)	0
Psychologist	1 (7.2%)	0	1 (16.7%)	0
**Specialties:**				
Obstetricians	2 (14.3%)	0	1 (16.7%)	1 (25%)
Neonatal physicians	7 (50%)	4 (100%)	1 (16.7%)	2 (50%)
Intensive care physicians	2 (14.3%)	0	2 (33.3%)	0
Paediatric neurologists	2 (14.3%)	0	1 (16.7%)	1 (25%)
Psychologist	1 (7.1%)	0	1 (16.7%)	0
Type of practice with very preterm children*:				
Perinatal period only	2 (14.3%)	0	1 (16.7%)	1 (25%)
Perinatal period + short-term care	8 (57.2%)	4 (100%)	3 (50%)	1 (25%)
Perinatal + short-term care + long-term follow-up	3 (21.4%)	0	2 (33.3%)	1 (25%)
Long-term follow-up only	1 (7.1%)	0	0	1 (25%)
**Duration of the Focusgroups** (minutes)	100 (60-120)	60	120	120

*Type of practice with extremely preterm children: “short-term care” = during hospitalisation; “long-term follow-up”= consultations for follow-up for more than 4 years with neurocognitive screening after 4 years.

### Content analysis

According to all the participants, the concept of HRQoL encompasses several aspects of life and is influenced by the environment in which we live (evaluation necessarily contextual: social, cultural, economical…). It is as a complex concept that can be limited to a consequence of one’s health. Although the definition of HRQoL was not clear according to the participants, a consensus emerged about the impact of health on various aspects of life, with particular importance assigned to family, social and school contexts. Parental evaluation of the HRQoL of children was deemed necessary (“*Parents are the mainjudges of their children*’*s lives*”) as assessment of the HRQoL of parents (“*The QoL of achild can be goodeven if the QoLof his or herparents is not”)*. This finding does not exclude the value of caregivers’ assessments (*“It is interesting toevaluate each person’s perception[parents, child, caregivers] toconfront his/her opinions”).* Because perceptions are inherently subjective, they can vary significantly; a neonatologist asked if a perception could be more valid than the other one and in which perception to trust. (*“What is the truth?”).*

Participants were confronted with the difficulty of HRQoL subjective and changing nature (i.e., that it is an adaptive process that changes as priorities change) (An intensive care physician said:*“Patients with a handicapcan have a goodQoL at one timeand a bad QoLat another period intheir lives, but itis the same thingfor everybody, with orwithout handicaps; it dependson children’s psychological evolution,their social integration, theconsciousness that they haveof their difficulties, theirfamily situation…”*).

One limitation reported by the participants was related to the interpretation of the quantitative values derived from the questionnaires, that asked the meanings of norms. Moreover, the FGs revealed an interest in conducting assessments measuring different dimensions of HRQoL (“*We can imagine thata child who hasa walking disorder isnot satisfied because hecannot play for sportswith his friends butat the same time,is able to playmusic, which would bebetter for him*”).

Health-related quality of life seems to be a potential informational tool: along with other factors within the framework of prematurity, HRQoL adds an additional dimension to evaluations of the EPC, especially when parents ask for concrete information about the future of their child (A neonatologist said: *“Parents want to knowwhat they are waitingfor with their child:How will they planhis/her school or educationallife? Could he/she makefriends? Could he/she playsports or engage incultural activities? What relationshipswill he/she have withothers? What will theduration of the diseasebe? How frequently willhospitalizations occur?”*). According to some neonatologists and the two paediatric neurologists, health-related quality of life evaluation could improve communication among families, children and caregivers, helping families anticipate problems and understand why and in what situations HRQoL can decline.

Knowledge about HRQoL studies and about the tools used to measure HRQoL was not precise, even when participants (particularly neonatologists) said that the QoL concept was sometimes used among “*ethical thinking staff*” in perinatal units, when they have to discuss withdrawing therapy.

There were no differences by gender, age or seniority, but perspectives varied by specialty and type of practice. Physicians who cared for EPC only during the perinatal period (i,e., obstetricians and intensive care physicians) said they did not take HRQoL into account. According to them, this notion is too subjective, depending of many factors and too evolutive to be taken into account. The application of this notion in the emergency perinatal contexts cannot be supposed. Conversely, physicians who provided long-term follow-up care for EPC (i,e., neonatal physicians and paediatric neurologists) indicated that considering HRQoL was essential, at the same time to improve the relation of care and the information. The paragraphs below illustrate this.

The vision of the HRQoL concept was slightly inconsistent with the themes discussed among the neonatal physicians. In reflecting on HRQoL, some were certain of its value for improving care and providing information. Others were more reserved, noting that HRQoL changes both over time and between patients (“*Responses to illness arehighly individual, with difficultyin generalising QoL data*”*),* thus limiting its utilization. Some neonatal physicians explained their hesitation and their difficulty in establishing standards for QoL, which cannot be generalizable. Quality of life could suggest a reflection of “life” and the notion of “quality”:“*What is a goodlife*?” with the “*idea of a judgementthat would depend onthe society in whichwe live*”.

Paediatric neurologists and neonatal physicians involved in long-term follow-up were conversely more aware of HRQoL methodology. They were more sensitive to the impact of prematurity on HRQoL both because they followed families more closely and because they were aware of the burden of care and the psychological, social and economic consequences. (A neonatologist said: “*To estimate the HRQoLof our patients, itis to estimate theecho of the pathologyin their daily life*.” A paediatric neurologist added: “*It can influence thecare, by estimating forexample the burden ofthe reeducations*”.)

Consideration of long-term outcomes was irrelevant to professionals involved in acute perinatal care (An obstetrician said: “*How can I justifythe delivery of anextremely preterm infant inthe context of anemergency if the datasay that QoL couldbe bad? The immediatestakes in certain urgentperinatal situations outweigh thelong-term potential QoL inchildren*…*It is impossible totalk to parents aboutdata such as QoLbecause it is aquestion of managing situationswith stakes in short-termsurvival, not of riskingfailure in the future.*”). HRQoL seemed to appear “*dangerous*” to some participants (An obstetrician said: “*it is not enoughto estimate QoL: itis necessary to knowwhat we are goingto do with thisevaluation*…*Can it influence medicaldecisions, knowing that thisconcept is evolutionary? Inprenatal staff, using QoLdata could favour terminationof pregnancy requests.”).* Because they provide care focused on patient survival rather than long-term qualitative outcomes, intensive care physicians believe that HRQoL could not represent a decision-making argument in terms of neonatal resuscitation, raising the issue of the “*sacredness of life*” versus the “*QoL*”. (An intensive care physician said: “*When a very pretermchild is born, theimmediate stake is notits long-term quality oflife, but mostly itssurvival*”.)

## Discussion

In this preliminary study, focus groups made appear themes of reflection classically told about HRQoL in other domains but not all in so clear a way (definition, interests and limits, ethical reflection) [13,14,24,25].

All the themes discussed hold considerable interest for the participants. These themes touched many dimensions: QoL as both a theoretical and practical issue, the physicians’ practical experience, that of the value as well as the limits to this type of approach. However, three sub-themes seemed particularly relevant to the group.

The first one of them concerns the definition of the quality of life.

The FGs’ participants seemed to have embraced the definition of HRQoL given by Eiser and Morse, identifying some key elements such as “subjectivity and multidimensional aspects” [26]. Although they recognized a subjective aspect, the participants indicated that a parental approach was necessary. This pragmatic attitude is in accordance with several studies in which parental evaluation was realized, especially with children less than six years old [5,6,8,27].

The second sub-theme concerns the limits of the use of quality of life concepts.

One of the limitations identified by users is that it seems difficult that the construct underlying the QoL is not constant at all ages of life of children and adolescents. The adaptive process relative to these specific periods of life are multiple referring indifferently to complex process of redefinition but also of recalibration of response or reprioritization of domains. The nature of HRQoL must be renegotiated throughout life [28]. Therefore, it seems illusory to imagine that what structures the QoL of each individual is invariable. For the participants, this evolution would prevent any standardized HRQoL evaluation. Because a child is continuously maturing, developing and changing, then the whole concept of HRQoL is invalid anyway.

Why would this “evolutionary” factor be a limitation for subjective measurement and not for objective measurement? One obstacle to using QoL measurement seems to be related to the implicit comparison with a standard or norm (perhaps because of a misunderstanding about the tools of measurement). Obviously, this subject is often reported on by experts who argue for the subjectivity of the measure used. However, in the field of so-called “non-objective” measures, many indicators are used to assess consensual aspects, such as intelligence quotient. Additionally, one might similarly question the value of several of our so-called objective measures, such as neurocognitive assessments, behaviour disorders, children’ size… The standards of certain objective criteria are not without problems. This issue raises the problem of the definition of the standard and its utilization. Canguilhem discussed the individualization of the standard in his criticism of the positivist determination of the normal and the pathological [29]. The “pathological” becomes an experience lived before being measured. Mistrust towards the objectivity of standards is, henceforth, a common problem in contemporary ethical culture. Using HRQoL standards does not mean defining a “normal life” but improving the everyday life of patients according to their situations [30,31].

Finally, the third sub-theme concerns decision-making with respect to QoL.

The main question addressed in the FGs was whether HRQoL assessments can assist in evidence-based decision-making. HRQoL seems to have a limited impact on perinatal guidelines. Globally, HRQoL is almost exclusively used as secondary criterion [17,32,33]. What would be the limit of this criterion if HRQoL data were available? There are many paradoxes. Participants said they were not familiar with HRQoL tools, but they knew the different aspects of the debate surrounding the topic with real practice attitudes. The differences in the evocation of QoL concept among “ethical thinking staffs” in perinatal units for withdrawing therapy (a way of clearing themselves of responsibility by taking into account at least one time what people could feel?), the lack of knowledge concerning evaluation methods and the fears expressed in the FGs do not position HRQoL as a criterion for making decisions in perinatal situations.

There were several limitations to our study. Participants were selected from one geographic region, and their experiences and opinions may not be generalizable. It is recognized in FG research that the recruited sample is not representative of the entire population but is rather a snapshot of those people participating in the study [34]. The presence of dominant participants and opinion leaders could have been a confounding factor; the experienced social psychologist controlled this issue. Some of the differences in perspectives that have appeared to be conflicts of opinions, can be explained by the varying professional and social positions of the participants. The sample size of the FGs should be discussed: to optimize group discussion, the size of the groups must be limited to avoid the creation of sub groups; at the same time, the groups must be large enough to allow for the emergence of a variety of controversial themes. The sample size and the limited number of FGs could appear as a limitation but saturation of information was nevertheless reached. Most authors support that adequate size of each group is between four and twelve individuals and the use of a minimum of three FGs with each type of participant; sufficient FGs have been conducted when no new information emerges from the dialogue of subsequent groups [34-36]. Focus Groups’ composition was not homogeneous because they were organized according to the availability of the participants. The heterogeneity of the participants allowed for the expression of different opinions about the subject. Paediatrician neurologists were included with perinatalology experts because they have experience in specialized EPC follow-up care. The participants were mostly female but it is a representation of the French medical reality and especially the profession of paediatrician, as it is described in the French review of social fields by Lapeyre and Le Feuvre [Unpublished observation: “Feminization of the medical profession and professional dynamics in the field of the health” *Revue française des affairessociales,* 2005, 1: 59–81] and in the report of the National Center of health professions’ demography [37].

Focus groups are used to explore topics on which little research has been conducted and have the advantage of enabling researchers to quickly identify the full range of perspectives held by participants [35]. Moreover, the interactional nature of FGs allows participants to clarify or expand on their contributions during the discussion in light of the points raised by the other participants.

## Conclusions

Our original, qualitative study provides preliminary results about physicians’ perspective on the health-related quality of life of extremely preterm children. This approach was never developed in the field of prematurity with well-knowed consequences on QoL. These results require to be confirmed on a larger representative sample including leaders of obstetrics, neonatal medicine, neonatal intensive care units of French University Hospitals as well french paediatrician neurologists. The themes and questions of this broad opinion survey will rest on the information issued from our preliminary interviews.

## Abbreviations

EPC: Extremely preterm children; HRQoL: Health-related quality of life; QoL: Quality of life; FG: Focus group; SC: Sophie Condopoulos; MAE: Marie-Ange Einaudi; MCS: Marie-Claude Simeoni.

## Competing interests

The authors declare that they have no competing interests.

## Authors’ contributions

MCS, PA and MAE participated in the conception and design of the study. SC and MAE analyzed the data. PA and MAE participated in the drafting of the article. All the authors contributed to a critical revision of the manuscript and made a substantial contribution to its content, and read and approved the final manuscript.
